# Quantitative Detection of Extra Virgin Olive Oil Adulteration, as Opposed to Peanut and Soybean Oil, Employing LED-Induced Fluorescence Spectroscopy

**DOI:** 10.3390/s22031227

**Published:** 2022-02-06

**Authors:** Ting Zhang, Yuyang Liu, Zhuoping Dai, Lihan Cui, Hongze Lin, Zejian Li, Kaihua Wu, Guangyu Liu

**Affiliations:** 1 School of Automation, Hangzhou Dianzi University, Hangzhou 310018, China; zt18196108@hdu.edu.cn (T.Z.); yuyangliu@hdu.edu.cn (Y.L.); 18196102@hdu.edu.cn (Z.D.); clh@hdu.edu.cn (L.C.); wukaihua@hdu.edu.cn (K.W.); g.liu@hdu.edu.cn (G.L.); 2 Zhejiang Key Laboratory of Design and Intelligence and Digital Creativity, College of Computer Science and Technology, Zhejiang University, Hangzhou 310027, China; zejianlee@zju.edu.cn

**Keywords:** fluorescence spectroscopy, extra virgin olive oil (EVOO), adulteration, LED-induced fluorescence, UV LED, pre-processing

## Abstract

As it is high in value, extra virgin olive oil (EVOO) is frequently blended with inferior vegetable oils. This study presents an optical method for determining the adulteration level of EVOO with soybean oil as well as peanut oil using LED-induced fluorescence spectroscopy. Eight LEDs with central wavelengths from ultra-violet (UV) to blue are tested to induce the fluorescence spectra of EVOO, peanut oil, and soybean oil, and the UV LED of 372 nm is selected for further detection. Samples are prepared by mixing olive oil with different volume fractions of peanut or soybean oil, and their fluorescence spectra are collected. Different pre-processing and regression methods are utilized to build the prediction model, and good linearity is obtained between the predicted and actual adulteration concentration. This result, accompanied by the non-destruction and no pre-treatment characteristics, proves that it is feasible to use LED-induced fluorescence spectroscopy as a way to investigate the EVOO adulteration level, and paves the way for building a hand-hold device that can be applied to real market conditions in the future.

## 1. Introduction

Known as the “Queen of Vegetable Oil” or “Liquid Gold”, olive oil has been involved in the diet of human beings for more than 4000 years. Abundant in unsaturated fatty acids, squalene, polyphenols, vitamins, and other nutrients, olive oil plays an important role in reducing the incidences of diet-related chronic diseases, cardiovascular diseases, and cancers [1]. Among various standards, extra virgin olive oil (EVOO) is the highest grade for its richness in nutrition, better flavor, and odor [1,2]. However, the yield of EVOO is low while the demand from the market is high, leading to adulteration and shoddy substitution occurrences in the market [3,4,5]. Therefore, a quantitative detection method that can help measure the EVOO adulteration level of blended oils is highly desirable.

It is feasible to classify edible vegetable oils with the help of an electronic nose [6], high-performance liquid chromatography (HPLC) [7,8], mass spectrometry [9], nuclear and magnetic resonance spectrometry (NMR) [10,11], etc. However, these methods have disadvantages, such as high equipment cost, long consuming time, tedious measurement process, and samples destruction, etc., which limit their application to blended oil detection in real life. Optical method has become one of the main approaches in food quality detection on account of its advantages: rapid, sensitive, and non-intrusive [3,12]. Absorption spectroscopy plays an important role in food adulteration detection based on the fact that different molecules have their unique absorption peaks. However, in the near infrared region, the absorption peaks of various substances overlap, which prevent precise prediction of adulteration level [13,14]. Mid infrared absorption spectroscopy is able to distinguish different oil samples accurately. The drawback of this method lies in the high cost of the equipment [15]. Raman spectroscopy suffers the influence of the fluorescence background, and the signal-to-noise ratio is often low [16]. Laser-induced fluorescence (LIF) spectroscopy employs laser with certain wavelength and strength to induce specific fluorescence signals, which act as “fingerprints” for classification. This technology has been applied to fields, such as EVOO adulteration [17,18,19] and tea classification [20], etc. However, the wavelengths that are affordable in the blue-green region are limited; common choices contain 532 nm from Nd:YAG lasers, as well as 405, 450, and 473 nm from semiconductor diode lasers. Recently, light-emitting diodes (LEDs) have dominated the illumination field due to the advantages of high brightness, long lifetime, and easy operation. With the rapid development of semiconductor technology, LEDs, with more commercially available wavelengths, can now be offered with decent light intensity from 370 to 470 nm. This facilitates the replacement of the exciting light source of LIF, resulting in what is called LED-induced fluorescence spectroscopy, which is expanding its use in various types of food detection applications [16,21,22]. Compared with LIF, LED-induced fluorescence spectroscopy requires less maintenance skills, smaller installation volume, and much lower cost, especially in the UV region.

In this paper, we use LEDs to induce the fluorescence signal of different adulterated EVOOs. The adulteration is conducted by peanut oil (PO) and soybean oil (SO), which are also common edible oils with much lower prices compared to EVOO. Blue LEDs with wavelengths of 396 nm, 402 nm, 414 nm, 441 nm, 451 nm, and 465 nm, as well as UV LEDs with wavelengths of 370 nm and 372 nm are utilized as the excitation light source to induce the fluorescence signals of blended oils, and the best wavelength is selected. The spectra of adulterated oil samples are recorded. Different pre-processing and regression methods are employed to build a prediction model between the actual adulteration condition and their fluorescence spectra, and their prediction results are compared. Our method proves that it is possible to detect the adulteration level using LED-induced spectroscopy, and the results pave the way for building a hand-held device that can be used in real market conditions in the future.

## 2. Materials and Methods

### 2.1. Samples

The EVOO, PO, and SO samples were bought from a local supermarket. The mixed oil samples were prepared by mixing PO or SO with EVOO at different levels. Nine gradients with doping concentrations from 10% to 90% and an interval of 10% were set, with each containing ten doping samples. Thus, in total, 180 doping samples were collected. Each sample mixture was prepared in a fused-quartz cuvette with a size of 50 × 14 × 50 mm, providing sufficient area for fluorescence signal excitation and collection. In order to ensure uniformity, all diluted samples were stirred for 10 min and tested immediately after preparation.

### 2.2. Apparatus of the Fluorescence System

**Figure 1:**
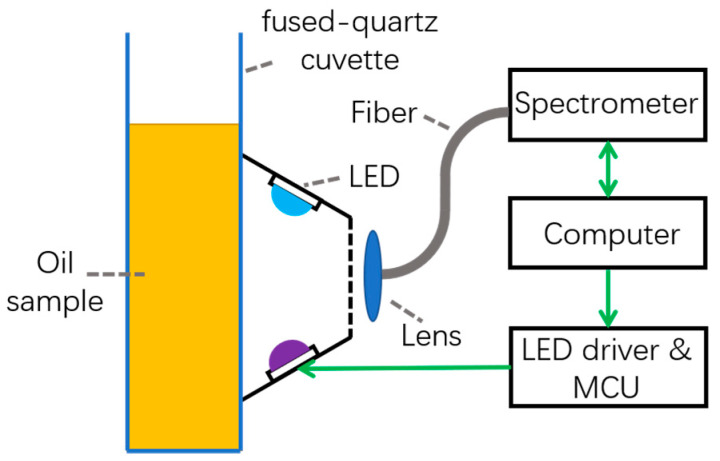
Schematic diagram of the fluorescence system.

The schematic diagram of the fluorescence system is shown in Figure 1. The LEDs were mounted on a ring structure with an incident angle of 60°. The incident angle was defined as the angle between the light direction and the surface of the oil sample. The excitation intensity was controlled by adjusting the output of a current power supply. The fluorescence signal generated by the oil sample was collected by a lens and focused into an optical fiber, which would lead the light into a spectrometer (FX2000, IdeaOptics, Shanghai, China) to transform the optical signal to digital signal, which would be finally processed by a computer. The computer was also in charge of LED alteration by sending commands to the micro control unit (MCU) of the LED driver. To reduce the influence of environmental factors on the experimental results, the whole data collection process was carried out in a dark environment. The fluorophores are sensitive to the wavelength of excitation light. To investigate the best excitation wavelength, eight LEDs with a central wavelength of 370 nm, 372 nm, 396 nm, 402 nm, 414 nm, 441 nm, 451 nm, and 465 nm were employed to excite the fluorescence of pure EVOO, PO, and SO samples, respectively. An acquisition time of 1 s was used. The excitation wavelength, which could induce the most fluorescence information, was chosen for further detection.

### 2.3. Data Pre-Processing Method

The data treatment was carried out by Python. Aiming to achieving a better training performance of the spectral data, pre-processing methods were utilized. Firstly, the environmental noise was collected after each measurement with the excitation LED turned off, and was subtracted from the previously obtained fluorescence spectrum. Subsequently, the Savitzky-Golay (S-G) smoothing method, which was realized by fitting successive subsets of adjacent data points with a two-degree polynomial and fitting window of 21 points [23], was employed to reduce high-frequency noise caused by the nonuniform light responsibility of each pixel on the CCD or CMOS of the spectrometer. Thirdly, four normalization methods, e.g., the standard normal variate and normalization with fluorescence peaks of three different regions were employed to eliminate the spectrum difference that becomes induced by a fluctuation of excitation light intensity as well as drifts of sample placement to improve the robustness of the model that would be built.

The standard normal variate (SNV) is a normal variate with a mean of 0 and standard deviation of 1. Light transmitted or reflected from inhomogeneous samples would cause some fluctuations behaved as noise in the spectra observed. SNV is suitable to reduce this fluctuation. The variation was processed as follows:

(1) $$Z_{i}=\frac{x_{i}-\bar{x}}{S}$$

where *Z_i_* is the variated intensity of the *i*th wavelength channel, *x_i_* is the intensity of the *i*th wavelength channel, $\bar{x}$ is the mean intensity of the spectrum, and *S* is the standard deviation of the spectrum.

Normalization was also employed to the spectra with three fluorescence peaks of different regions, i.e., the yellow-green region, the red region, and the whole spectrum, respectively. The fluorescence peak in the red region is supposed to be linked with chlorophyll—mainly, chlorophyll *a*. The fluorescence peak in the yellow-green region may be owed to riboflavin, vitamin E, or flavins [24,25]. The normalization process was as follows:

(2) $$Z_{i}=x_{i}/\max(x_{region})$$

where *region* can be 500~550 nm, 650~700 nm, or 500~700 nm.

### 2.4. Data Regression and Evaluation Method

Two regression methods, including principal component analysis (PCA) combined with multiple linear regression (MLR) as well as partial least square regression (PLSR), were used to process the spectral data after pre-processing.

PCA is one of the most useful multivariate analysis tools. The major objective of PCA is to establish the linear combinations of the original variables and extract the useful information from the original data by reducing the dimensionality of original data. The variables can be reduced by eliminating the overlapping components, and the most important information contained in the original data is retained by PCA. MLR displays a direct relationship between the dependent variable Y and independent variable X. In MLR analysis, the mean of the dependent variable Y relies on X. The MLR equation can be used to incorporate more than one independent variable with a single response variable [26].

PLSR is a standard method for analyzing high-dimensional and multicollinear data, and was thus employed to model the relationship between the fluorescence spectra and the dilution concentration [27]. PLSR takes both the predictor and response variables into account, and assumes that the investigated system or process is driven by a set of underlying latent variables (LVs). It searches for linear combinations of predictor variables X that maximize the covariance between the LV and the response y. This procedure is iteratively conducted using a deflation scheme to ensure the mutual orthogonality of the LVs.

To evaluate the accuracy of predictions, the coefficient of determination (R^2^) as well as the root mean square error (RMSE) was utilized. The leave-one-out cross validation method was adopted to build the validation set.

## 3. Results and Discussion

### 3.1. Fluorescence Spectra Excited by LEDs with Different Wavelengths

**Figure 2:**
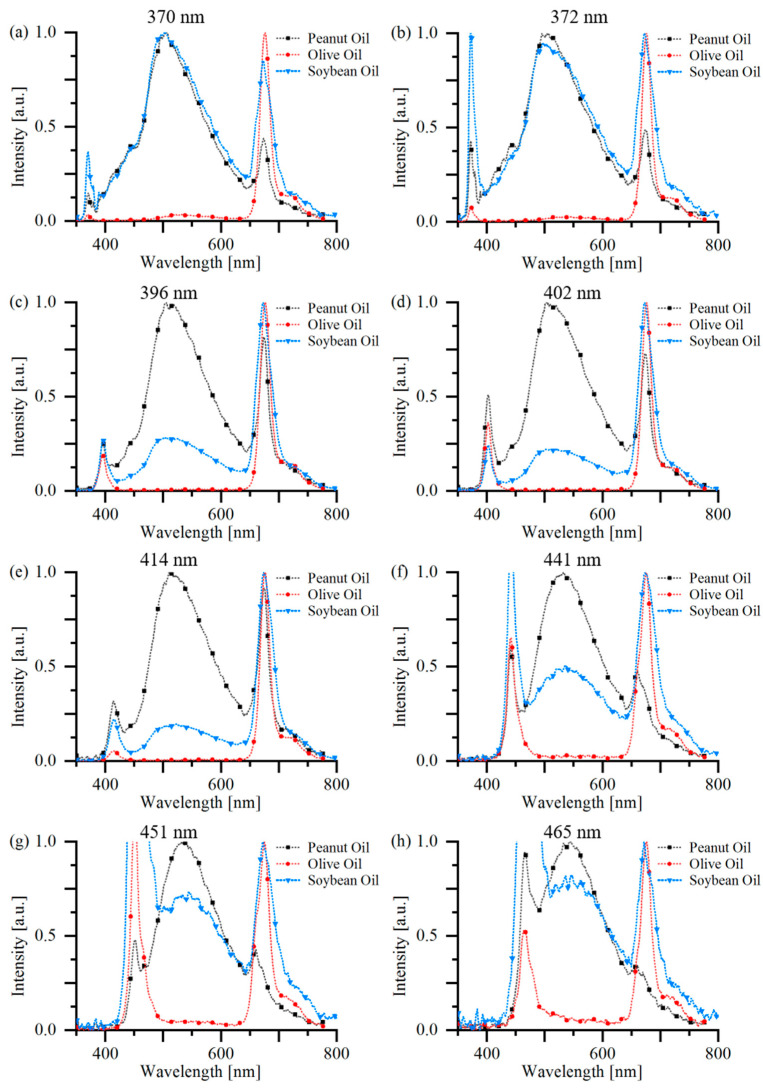
Fluorescence spectra of pure EVOO, PO, and SO, excited by LEDs with wavelength of (**a**) 370 nm, (**b**) 372 nm, (**c**) 396 nm, (**d**) 402 nm, (**e**) 414 nm, (**f**) 441 nm, (**g**) 451 nm, and (**h**) 465 nm, respectively.

The fluorescence spectra of different pure oil samples under excitation of LEDs with various wavelengths are depicted in Figure 2. These spectra were smoothed and normalized to their maximum amplitudes of 500~700 nm.

As can be observed from Figure 2, all three kinds of oil contain chlorophyll, which emits the fluorescence peak around 685 nm, as well as a weaker fluorescence peak around 740 nm. Both PO and SO have similar fluorescence peaks around 505 nm, which can be excited by LEDs with wavelength from 370~465 nm, indicating that the fluorophores may be the same. The yellow-green fluorescence peak of EVOO is rare, and can only be observed when excited by LEDs of UV or 441 nm, 451 nm, and 465 nm. The latter two are similar to previous publications, which found fluorescence peaks in this region with an excitation wavelength of 473 nm [17] or 450 nm [18]. However, the light sources used in [17,18] were lasers, which are narrow in bandwidths (typically less than 1 nm) compared to that of LEDs (typically ten to twenty nanometers). Another phenomenon that can be observed is that the longer the central wavelength, the wider the bandwidth [22]. Thus, though lasers of wavelength larger than 440 nm can be utilized for oil adulteration detection, LEDs of this band are not suitable, because their own spectrum may overlap with the fluorescence spectrum. Thus, the excitation wavelength of 372 nm was selected, as it can generate not only the fluorescence of chlorophyll but also substances emitting fluorescence in the 400~550 nm region of all three edible oils, thus offering the most sufficient information for further analysis [28]. Despite the obvious fluorescence peaks located at 505 nm of SO and PO, and 525 nm of EVOO, a third fluorescence peak can be obtained by comparing fluorescence signals of PO or SO at a wavelength range of 400~450 nm of Figure 2a,c, with a central wavelength of 430 nm, which might relate with trans-β-carotene [29]. The origin of fluorescence signals in the 450~550 nm region is complex; possible contributors include vitamins A, K, and D, and NADH, NADPH, and flavins [24].

### 3.2. Regression Results

**Figure 3:**
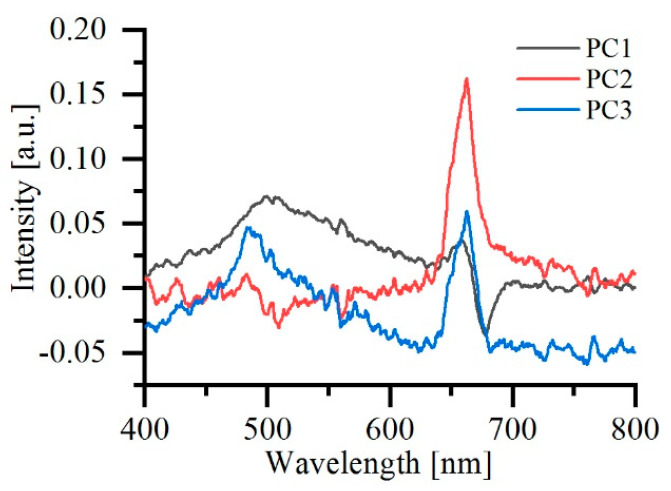
Diagram of PC1, PC2, and PC3 after dimensionality reduction by PCA.

The number of principal components (PCs) was determined by a scree or elbow test [17], and in total seven PCs were selected, which explained 99.4% of the total variance. The first three PCs were depicted in Figure 3. As can be observed, the first PC has a peak around 505 nm, which was correlated with the fluorescence peaks appearing in the PO and SO. A sudden drop appeared around 680~700 nm, which provides a shift of the chlorophyll fluorescence peak. Both the second and the third PCs show the chlorophyll fluorescence peak at around 685 nm. However, the second PC has a negative peak around 505 nm and the third PC has a positive peak around 480 nm, which indicate the concentration relations of the corresponding fluorophores with chlorophyll *a*. In addition, the negative part of PC2 from 400 nm to around 650 nm may also be correlated with the absorption spectrum of chlorophyll *a*, which indicates that while PO or SO will compete with EVOO to absorb the UV light and hence reduce the fluorescence intensity of EVOO, their existence may also enhance the fluorescence peaks of chlorophyll *a* of EVOO because their fluorescence provides more energy for the chlorophyll *a* to absorb.

The concentration of blended oil samples was predicted by two regression methods combined with four pre-processing methods. The prediction results were evaluated by R^2^ and RMSE, and are shown in Table 1. As a whole, SNV offers the best accuracy in all four pre-processing methods, followed by Norm_500~550,_ Norm_500~700_. The prediction accuracy of Norm_650~700_ is the worst. An explanation can be given that for samples with dominant composition, say, 90% EVOO with 10% SO, their fluorescence of the minor composition is weak. If the normalization was based on the weak fluorescence region, the noise was amplified, thus leading to a lower prediction result. As for the regression method, PLSR provides better prediction accuracy than the PCA + MLR method, which is because PLSR takes both the variables and response variables to build the regression model.

**Figure 4:**
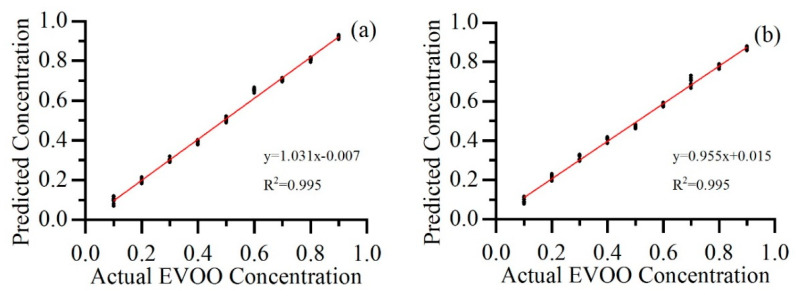
EVOO blended with (**a**) SO and (**b**) PO, with adulteration concentrations ranging from 10% to 90%.

The prediction results employing PLSR combined with the SNV method of EVOO diluted by SO and PO are visualized in Figure 4a,b, respectively. EVOO with concentrations from 10% to 90% and an interval of 10% are tested. Both linearities achieve R^2^ of 0.995 employing the same model, which proves the feasibility of the selected UV LED and the proposed pre-processing and regression method as a way for quantitative detection of extra virgin olive oil adulteration.

## 4. Conclusions

In this paper, an optical method based on LED-induced fluorescence technology and spectral analysis technology to identify the doping ratio of EVOO over edible vegetable oil is proposed. LEDs are employed as the light source to induce fluorescence from oil samples. Various wavelengths from UV to blue are tested, and the UV LED with the central wavelength of 372 nm is proven to be the one that can generate the most fluorescence signals, and is selected as the excitation light source. Different preprocessing methods, e.g., SNV, and the normalization of different wavelength bands are examined for evaluation accuracy combined with different regression algorithms. The SNV is proven to have the highest prediction accuracy. It can be concluded that the SNV is better than the normalization of different wavelength bands, and the PLSR provided better prediction accuracy than the PCA combined with MLR method. The best R^2^ retrieved by SNV and PLSR reaches 0.9951 for all doping samples, while its RMSE is 0.02236.

This experiment paves the way for building a handhold LED-induced fluorescence instrument to non-instructively detect the adulteration level of edible oil. The model we built should work to predict the adulteration level if the blended oil only consists of EVOO and SO or PO. However, towards adulteration level detection in real life, the following problems have to be solved. Firstly, the bottle material may influence the transmission of the excitation light as well as the fluorescence spectrum, making the field test even harder. Secondly, oil mixtures of more than two kinds often happens, including not only PO or SO, but also corn oil or rapeseed oil. A prediction model that is feasible for more oils is desired. We will try to improve the robustness of our technique in the future by building a handheld device that is useful in our daily life. Future works include involving more edible oils to build a more applicable prediction model, and detecting more excitation wavelengths and algorithms for edible oil adulteration detection.

## Figures and Tables

**Table 1:** Performance measurements of PCA + MLR and PLSR model for the prediction of EVOO concentration, using four different pre-processing methods. The best performance of each regression method is highlighted.

Regression Method	Pre-Processing	PCs or LVs	R^2^	RMSE
PCA + MLR	SNV	7	**0.9907**	0.0325
	Norm_500~550_	7	0.9897	0.0342
	Norm_650~700_	7	0.9564	0.0703
	Norm_500~700_	7	0.9614	0.0662
PLSR	SNV	7	**0.9951**	0.0236
	Norm_500~550_	7	0.9949	0.0241
	Norm_650~700_	7	0.9830	0.0439
	Norm_500~700_	7	0.9871	0.0382

## Data Availability

Data available on request from the corresponding author.
